# Single-Round Infectious Particle Antiviral Screening Assays for the Japanese Encephalitis Virus

**DOI:** 10.3390/v9040076

**Published:** 2017-04-10

**Authors:** Chien-Yi Lu, Mann-Jen Hour, Ching-Ying Wang, Su-Hua Huang, Wen-Xiang Mu, Yu-Chun Chang, Cheng-Wen Lin

**Affiliations:** 1 Department of Medical Laboratory Science and Biotechnology, China Medical University, Taichung 40402, Taiwan; cylu0424@gmail.com (C.-Y.L.); spirit1126@hotmail.com (C.-Y.W.); z5116333@gmail.com (W.-X.M.); cyj770603@gmail.com (Y.-C.C.); 2 School of Pharmacy, China Medical University, Taichung 40402, Taiwan; mjhou@mail.cmu.edu.tw; 3 Department of Biotechnology, Asia University, Taichung 41354, Taiwan; shhuang@asia.edu.tw

**Keywords:** Japanese Encephalitis virus, replicon, single-round infectious particle, green fluorescent protein, flow cytometry, antiviral potency

## Abstract

Japanese Encephalitis virus (JEV) is a mosquito-borne flavivirus with a positive-sense single-stranded RNA genome that contains a big open reading frame (ORF) flanked by 5′- and 3′- untranslated regions (UTRs). Nearly 30,000 JE cases with 10,000 deaths are still annually reported in East Asia. Although the JEV genotype III vaccine has been licensed, it elicits a lower protection against other genotypes. Moreover, no effective treatment for a JE case is developed. This study constructed a pBR322-based and cytomegaloviruses (CMV) promoter-driven JEV replicon for the production of JEV single-round infectious particles (SRIPs) in a packaging cell line expressing viral structural proteins. Genetic instability of JEV genome cDNA in the pBR322 plasmid was associated with the prokaryotic promoter at 5′ end of the JEV genome that triggers the expression of the structural proteins in *E. coli*. JEV structural proteins were toxic *E. coli*, thus the encoding region for structural proteins was replaced by a reporter gene (enhanced green fluorescent protein, EGFP) that was in-frame fused with the first eight amino acids of the C protein at N-terminus and the foot-and-mouth disease virus (FMDV) 2A peptide at C-terminus in a pBR322-based JEV-EGFP replicon. JEV-EGFP SRIPs generated from JEV-EGFP replicon-transfected packaging cells displayed the infectivity with cytopathic effect induction, self-replication of viral genomes, and the expression of EGFP and viral proteins. Moreover, the combination of JEV-EGFP SRIP plus flow cytometry was used to determine the half maximal inhibitory concentration (IC50) values of antiviral agents according to fluorescent intensity and positivity of SRIP-infected packaging cells post treatment. MJ-47, a quinazolinone derivative, significantly inhibited JEV-induced cytopathic effect, reducing the replication and expression of JEV-EGFP replicon in vitro. The IC50 value of 6.28 µM for MJ-47 against JEV was determined by the assay of JEV-EGFP SRIP infection in packaging cells plus flow cytometry that was more sensitive, effective, and efficient compared to the traditional plaque assay. Therefore, the system of JEV-EGFP SRIPs plus flow cytometry was a rapid and reliable platform for screening antiviral agents and evaluating antiviral potency.

## 1. Introduction

Japanese Encephalitis virus (JEV), a member of mosquito-borne flaviviruses, is an enveloped, positive-sense single-stranded RNA virus [1]. Like other flaviviruses including dengue virus, West Nile virus, and Zika virus, JEV genome has an only open reading frame (ORF) encoding a polyprotein consisting of structural proteins (capsid (C), membrane (prM/M), and envelope (E)) and non-structural proteins (NS1, NS2A, NS2B, NS3, NS4A, NS4B, and NS5) [2,3]. C protein contains positively charged residues, enabling to bind viral RNA and form a ribonucleoprotein complex [4]. The signal peptide of prM/M protein is sliced by a cell protease during maturation [5]. E protein (E) is responsible for virus attachment and membrane fusion and is also the crucial antigen to inducing neutralizing antibodies [6,7]. Non-structural proteins NS1, NS2A, NS2B, NS3, NS4A, NS4B, and NS5, are responsible for viral replication [1,2,3]. NS1 protein is associated with the initial negative-strand RNA synthesis in the early stage replication, secreted to modulate the host immune response [8,9]. NS2A is a membrane associated protein that participates in virus replication and assembly [10]. NS2B is a co-factor to activate the proteolytic activity of NS3 protein, which contains protease, helicase, and NTPase domains [11,12]. A hydrophobic protein NS4A is also related to viral replication [13]. NS4B and NS5 can block interferon signaling [14]; moreover, NS5 is recognized as an RNA-dependent RNA polymerase and methyltransferase [15]. JEV infection might lead to developing permanent neurologic or psychiatric sequelae, including memory loss, behavior disturbances, impaired cognition, convulsions and paralysis, and severe central nervous system damage [16]. JEV is predominant in Southeast Asia and East Asia due to the distribution of *Culex* species. Although inactivated JEV genotype III vaccines have been invented and introduced in many countries, more than 30,000 JE cases including 10,000 deaths are annually reported in these areas [17]. Recently, genotype I JEV has appeared in Vietnam, Taiwan, China, and Korea, potentially becoming the predominant genotype in these countries [1,18]. Noticeably, the inactivated JEV genotype III vaccine elicited a low protective immunity against the JEV genotype I [19]. Nowadays, there is still not an effective treatment yet for JE cases; the rapid and rational screening platforms are necessary for efficiently developing anti-JEV agents.

Replicons of positive-strand RNA viruses, such as hepatitis C virus, coronavirus, dengue virus, West Nile virus, and JEV are used to screen antiviral compounds and examine antiviral mechanisms [20,21,22,23,24,25,26]. Replicons contain a partial genome, including cis-acting elements in the 5′- and 3′-ends as well as the genes of all non-structural proteins under the control of cytomegalovirus (CMV) immediate-early promoter. The CMV promoter driven viral RNA subgenomes enable self-replication in transfected cells but have no infectious particles [27,28]. Moreover, reporter genes like green fluorescent protein (GFP) and firefly luciferase are in-frame cloned into viral subgenomes in the replicons, and quantitative analysis of the reporter expression becomes a measurable assay to monitor the virus replication. DNA-launched replicons are more convenient than RNA-launched replicons required producing viral RNA subgenomes using in vitro transcription assays [29]. Because they are lacking the structure protein coding region, both types of virus replicons transiently self-replicate in cells post transfection and could not generate the infectious particles for analyzing the early stages of viral replication [30]. Subsequently, DNA-launched replicons are transfected into the cells carrying the recombinant plasmid encoding the viral structural proteins, which express structural proteins and self-replicate viral subgenomes to generate single-round infectious particles (SRIP). Several flavivirus replicon-based SRIPs including JEV, dengue virus, West Nile virus, and tick-borne encephalitis virus have been generated, demonstrating the immunogenicity of virus-like particles [31,32,33,34,35,36].

**Figure 1:**
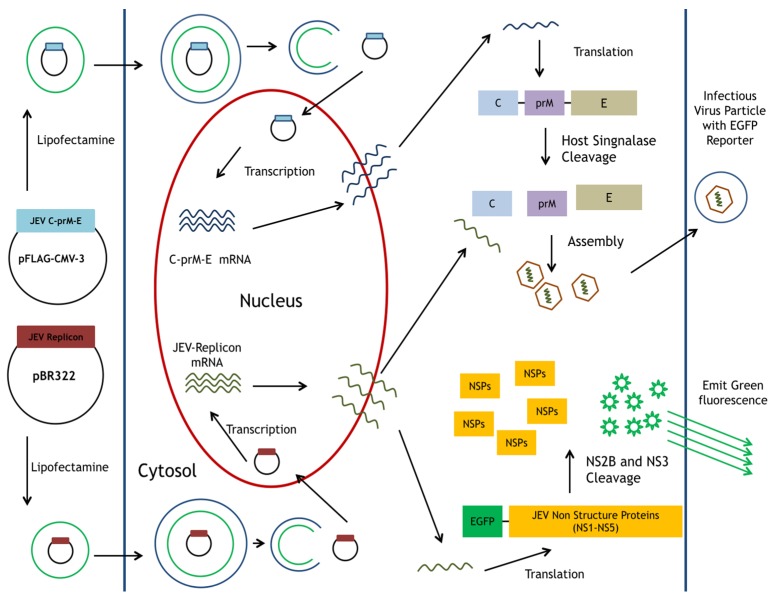
Schematic diagram of the production for JEV-EGFP SRIPs in pBR322-based JEV replicon-transfected packaging cells expressing JEV structural proteins. A reporter gene EGFP was the functional marker for the replication of replicon and SRIP in vitro.

Most reports recognize the replicon-based SRIPs as a suitable system for the development of safer vaccines against flavivirus infection. The replicon-based SRIPs containing the reporter genes of such fluorescent proteins could be more applicable as a rapid and real-time screening for the antiviral agents, as well as directly quantitate the antiviral activity under the condition without plaque-based assays. This study intends to establish a safe and reliable platform for screening antiviral agents and investigating antiviral mechanisms using JEV replicon-based SRIPs and flow cytometry (Figure 1). The reporter enhanced green fluorescence protein (EGFP) was fused with a foot-and-mouth disease virus (FMDV) 2A peptide, and then cloned and in-frame fused with NS1~5 coding regions in pBR322 plasmid-based JEV-EGFP replicon (named as pBR322-JEVrep-EGFP). Subsequently, JEV-EGFP SRIPs were generated in the cells co-transfected with pBR322-JEVrep-EGFP and pFlag-CMV3-CprME. The infectivity of JEV-EGFP SRIPs was characterized by the replication of viral subgenomes and the expression of EGFP as well as viral proteins using real-time PCR and fluorescent microscopy. Finally, the antiviral screening platform was performed according to comparing the fluorescence intensity of EGFP in SRIP-infected cells with and without treatment by flow cytometry. Accuracy and reproducibility of the half maximal inhibitory concentration (IC50) of anti-JEV agent MJ-47 by JEV-EGFP SRIP plus flow cytometry were further evaluated using plaque-reduction assay.

## 2. Methods and Materials

### 2.1. Cells and Virus

Human medulloblastoma TE671 cells were cultured in minimum essential medium (MEM, GE Healthcare Life Sciences, Pittsburgh, PA, USA) with 2% fetal bovine serum (ThermoFisher, Waltham, MA, USA), glutamine, pyruvate, and penicillin/streptomycin at 37 °C in an atmosphere containing 5% carbon dioxide. JEV T1P1 strain (GenBank: AF254453.1) used in this study was propagated in BHK-21 cells, as described in a prior report [36].

### 2.2. Preparation of pBR322-Based JEV-EGFP Replicon and pFlag-CMV3-CprME

**Figure 2:**
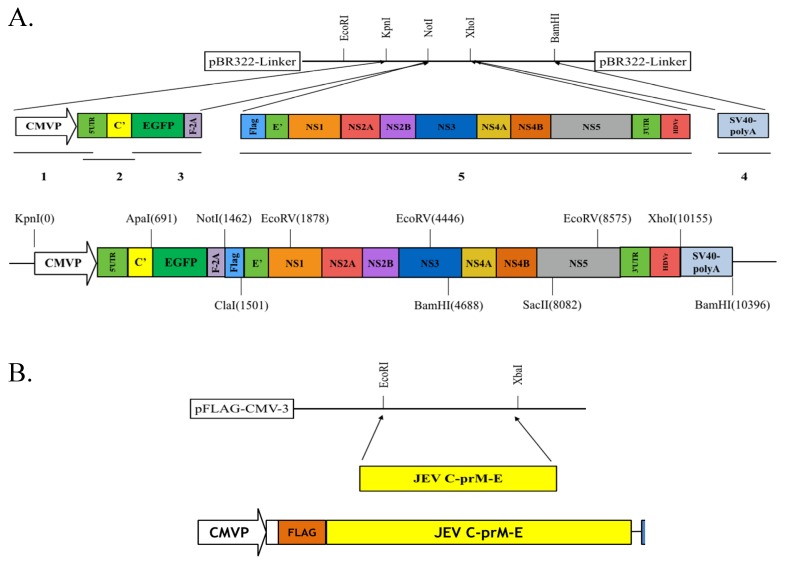
Construction of pBR322-based JEV replicon and pFlag-CMV3-CprME. (**A**) The DNA fragments of CMV promoter, JEV genome cDNA, EGFP and SV40-polyA were amplified using PCR, and then assembled into the indicated restriction sites of the pBR322 plasmid; (**B**) The cDNA sequence encoding structural proteins was amplified using PCR and cloned into the pFlag-CMV3 with EcoRI and XbaI double digestion. The detailed procedure was described in the section of Materials and Methods.

Plasmid pBR322-Linker was derived from pBR322 (ThermoFisher, Waltham, MA, USA) by the insertion of a 32-bp nucleotide linker containing EcoRI, KpnI, NotI, XhoI, and EcoRI into EcoRI/BamHI sites of pBR322 (Figure S1). Resultant pBR322-Linker was used as the vector for assembling 5 fragments of JEV-EGFP replicon consisting of five nucleotide (nt) fragments, including CMV immediate-early promoter, nt 1–119 of JEV T1P1 strain (GenBank: AF254453.1), EGFP/FMDV 2A peptide, nt 2388–10,970 of T1P1 strain/hepatitis delta virus ribozyme (HDVr), and SV40 polyA (Figure 2A). Each fragment was amplified using Platinum^®^ PCR Supermix High Fidelity reaction (Life Technology, Carlsbad, CA, USA) with indicated primer pairs and templates listed in Supplemental Table S1. Forward primer of Fragment 5 has an extra nucleotide sequence for Flag tag and ClaI restriction site. Reverse primers of Fragments 3 and 5 contain extra nucleotide sequences for FMVD 2A peptide and HDVr, respectively. The in-frame fusion of Fragments 1 and 2 were produced using a jumping PCR method (Figure S2), and cloned into KpnI and NotI sites of pBR322-Linker as pBR322-F1F2. Since a unique ApaI restriction site at nt119 of JEV, Fragment 3 was ligated with pBR322-F1F2 after ApaI and NotI double digestion, as pBR322-F1F2F3. Subsequently, Fragments 4 and 5 were cloned into XhoI/BamHI and NotI/XhoI sites of pBR322-F1F2F3. The assembled JEV-EGFP replicon with these five fragments was propagated in *E. coli* DH5α cells, and then verified by restriction enzyme analysis plus sequencing.

### 2.3. Functional Analysis of JEV-EGFP Replicon Using RT- PCR and Immunofluorescent Staining

For examining the synthesis of positive- and negative-sense RNA subgenomes, total RNAs of the transfected TE671 cells with JEV-EGFP replicon were extracted using PureLink Mini Total RNA Purification Kit (ThermoFisher, Waltham, MA, USA), reverse transcripted into cDNA with specific-capture primers, and followed by measuring positive- and negative-sense RNA subgenomes using SYBR Green-based real time PCR with JEV-specific primer pairs (Figure S3). Relative levels of RNA subgenomes were normalized by glyceraldehyde 3-phosphate dehydrogenase (GAPDH), described in a prior report [36]. To explore the expression of EGFP and JEV proteins, transfected cells were rinsed once with PBS, fixed with 4% formaldehyde in PBS at room temperature for 30 min, and permeabilized 0.1% Triton X-100, 10% BSA in PBS at room temperature for 1 hr. After blocking with 5% bovine serum albumin in PBS, permeabilized cells were stained using primary rabbit polyclonal antibodies, including anti-JEV E protein, anti-JEV NS3, and anti-JEV NS4B (GeneTex, Inc., Irvine, CA, USA), followed by the incubation with secondary AF546 goat anti-rabbit IgG (ThermoFisher, Waltham, MA, USA) in dark box for 2 h. After washing with PBS, stained cells were photographed using the immunofluorescence microscopy (Olympus, BX50, Tokyo, Japan).

### 2.4. Generation of a TE671 Cell Line Expressing C, prM/M, and E Proteins for the Production of JEV-EGFP SRIPs

**Figure 3:**
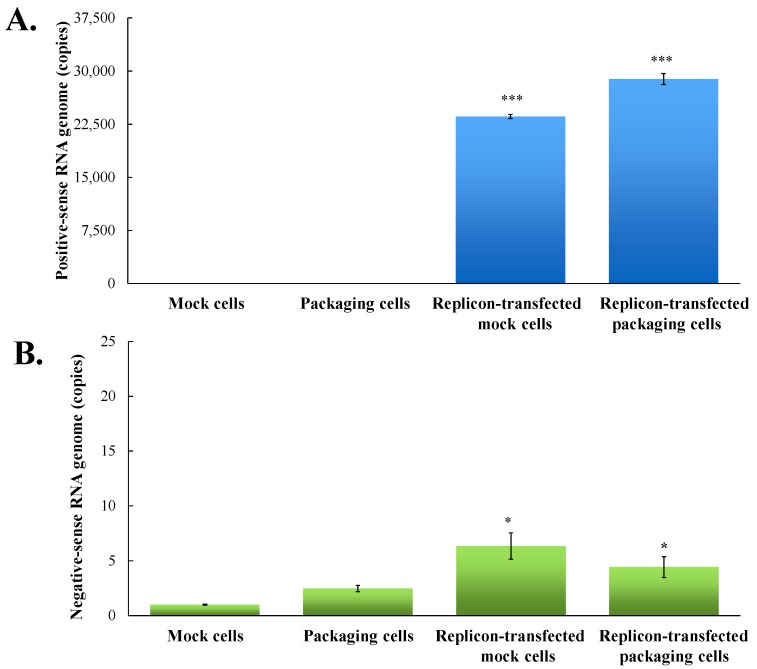
Quantitative analysis of positive- and negative-sense JEV subgenomes using strand-specific real time RT-PCR. Total RNAs were extracted from mock cells, packaging cells, replicon-transfected mock cells and replicon-transfected packaging cells; relative copy numbers of positive- (**A**) and negative-sense (**B**) subgenomes were quantitated using real-time PCR, and then normalized by GAPDH mRNA. *, *p*-value < 0.05; ***, *p*-value < 0.001 compared with mock cells.

To construct the recombinant plasmid for expressing JEV structural proteins in mammalian cells, the coding region for JEV structural proteins was amplified using PCR, and then cloned into EcoRI and XbaI sites of the expression plasmid pFlag-CMV3 (Figure 2B and Figure 3B). Resultant plasmid pFlag-CMV3-CprME was analyzed using restriction enzyme analysis and sequencing. All primers for sequencing were listed in Supplemental Table S2. No mutation in pFlag-CMV3-CprME was detected. To create a packaging cell line expressing JEV structural proteins, TE671 cells were grown to 90% confluence in a six-well plate, and then transfected with 4 µg of pFlag-CMV3-CprME using Lipofectamine LTX (Invitrogen, Carlsbad, CA, USA) according to the manufacturer’s guidelines. The transfected cells were treated with 500 µg/mL of G418 24 h post transfection; a stable transfected cell line was established after a 10-day-selection, in which the expression of JEV structural proteins was validated by immunofluorescence staining. Next, the packaging cell line was grown to 90% confluence, and then transfected with 4 µg of pBR322-JEV-EGFP replicon using Lipofectamine LTX. JEV-EGFP SRIPs were collected from the cultured media and lysate supernatant of transfected packaging cells. Finally, viral subgenome synthesis and viral protein expression in infected cells were examined using real time RT-PCR and immunofluorescent staining, as described above.

### 2.5. Infective Activity Assay of JEV-EGFP SRIPs Using Flow Cytometry

To determine the infectivity of JEV-EGFP SRIPs, CprME-packaging cells were infected with SRIPs at 10 TCID_50_, harvested 36 h post infection, and washed with PBS. After centrifuging at 8000 rpm for 3 min, cell pellets were dissolved with 100 μL PBS, and then fixed in 1 mL 70% alcohol at −20 °C overnight. After washing twice with PBS the next day, over 10,000 resuspended cells were analyzed by BD FACSAria (Becton Dickinson, Franklin Lakes, NJ, USA) with excitation at 495 nm and emission at 520 nm. Mock-infected CprME packaging cells served as negative controls and their fluorescent intensity was set as the threshold. The results were recorded by a ratio of EGFP-positive cells to total cells and the green fluorescent intensity of positive cells, respectively.

### 2.6. Synthesis of Compound MJ-47 

Compound MJ-47 (Methyl 2-((2-(3-methoxyphenyl)-6-(pyrrolidin-1-yl)quinazolin-4-yl)oxy)acetate) was synthesized based on a lead compound MJ-38 (Figure S4), an antineoplastic quinazolinone derivative [37], and then introduced a functional group, such as alkoxyl or aniline moiety, to the 4-position of quinazolinoe for decreasing cytotoxicity. HMJ-38 (0.60 g, 1.87 mmol), synthesized according to the published procedure [37], was dissolved in 30 mL *N*,*N*-dimethylformamide (DMF) and treated with sodium hydride (NaH) (60% dispersion in paraffin oil, Merck) at 50 °C for 30–60 min. Methyl chloroacetate (0.30 g, 2.81 mmol, Lancaster) was added to the above suspension with stirring at 50 °C for about 2 h. The mixture was poured into ice water (200 mL) and then filtered to collect the brown precipitate. The precipitate was washed with water, dried, and purified by column chromatography (silica gel; dichloromathane/*n*-hexane) and recrystallization with 95% ethanol to afford MJ-47 (yield 0.63 g (85.7%); brown crystals). The identity and purity of MJ-47 were confirmed by nuclear magnetic resonance spectroscopy and mass spectrum. Melting points were determined with a Yanaco MP-500D melting point apparatus and are uncorrected. Nuclear magnetic resonance (NMR) spectra were obtained on a Bruker Avance DRX-500 NMR spectrometer (Waltham, MA, USA) in Dimethyl sulfoxide (DMSO)-*d*_6_. The following abbreviations are used: *s*, singlet; *d*, doublet; *dd*, double doublet; *t*, triplet; *q*, quartet; *m*, multiplet; and *br*, broad. Mass spectrometry (MS) spectra were measured with a Finnigan/Thermo Quest MAT 95XL instrument (Waltham, MA, USA). Elemental analyses (C, H, and N) were performed on a Perkin-Elmer 2400 Series II CHNS/O analyzer (Waltham, MA, USA) or Elementar vario EL III Heraeus CHNOS Rapid F002 (Langenselbold, Hesse, Germany) and the results were within ±0.4% of the calculated values. Melting point: 165 °C; ^1^H NMR (DMSO-*d*_6_) δ 2.02–2.04 (4H, *m*, CH_2_CH_2_NCH_2_CH_2_), 3.39–3.41 (4H, *m*, CH_2_NCH_2_), 3.71 (3H, *s*, COOCH_3_), 3.85 (3H, *s*, OCH_3_), 5.23 (2H, *s*, OCH_2_CO), 6.89 (1H, *d*, *J* = 2.5 Hz, H-4′), 7.04 (1H, *dd*, *J* = 2.5, 8.0 Hz, H-5′), 7.41 (1H, *d*, *J* = 8.0 Hz, H-6′), 7.43 (1H, *s*, H-2′), 7.85 (1H, *d*, *J* = 8.0 Hz, H-7), 7.89 (1H, *s*, H-5), 7.95 (1H, *d*, *J* = 8.0 Hz, H-8) ppm; ^13^C NMR (DMSO-*d*_6_) δ 25.55, 48.04, 52.35, 55.57, 63.65, 98.91, 112.60, 116.11, 116.18, 119.99, 123.21, 129.19, 130.06, 139.54, 144.07, 146.84, 153.64, 159.99, 163.95, 169.51 ppm; MS (ESI) *m*/*z* 393; Anal. (C_22_H_23_N_3_O_4_): C, H, N.

### 2.7. Cytotoxicity of Compound MJ-47 with a MTT Assay

Cytotoxicity of MJ-47 to TE671 cells was measured using MTT assay. Cells were treated with MJ-47 (1, 10, 20, 50, and 100 μM) in quadruplicate at 37 °C in 5% CO_2_ for 48 h, incubated with 10 μL of 5 mg/mL MTT solution for 4 h, and then dissolved by DMSO (100 μL/well). Absorbance (OD_570–630_) was detected using a micro-ELISA reader. The survival rate was calculated as a ratio of OD_570–630_ of treated cells to OD_570–630_ of mock cells. Cytotoxic concentration giving 50% (CC_50_) was calculated by a computer program, as described in a prior report [36].

### 2.8. Inhibitory Assays of Cytopathic Effect and Replicon-Driven Replication by MJ-47

TE671 cells were infected with JEV at a multiplicity of infection (MOI) of 0.1 and simultaneously treated with MJ-47 (0, 1, 10, 20, and 50 μM). The photos of JEV-induced cytopathic effect in MJ-47-reated infected cells were taken by inverted microscope 36 and 48 h post infection. Moreover, mock TE671 cells were infected with JEV-EGFP SRIPs at 10 TCID_50_ and treated with MJ-47 4 h post transfection. The expression of EGFP and viral proteins as well as the synthesis of positive- and negative-sense RNA subgenomes in MJ-47-reated infected cells were determined using fluorescent microscope, immunofluorescent staining and real-time RT-PCR assays, as described above.

### 2.9. A Combination Assay of JEV-EGFP SRIP and Flow Cytometry for IC50

JEV CprME-expressing packaging cells were infected with JEV-EGFP SRIPs at 10 TCID_50_ in the presence and absence of MJ-47. After 36-h incubation, treated and infected cells were washed trypsinized, collected by the centrifugation at 2000 rpm for 3 min, fixed in 1 mL 70% alcohol at −20 °C overnight, and then were analyzed by BD FACSAria (Becton Dickinson) with excitation at 495 nm and emission at 520 nm. IC50 values were calculated according to a change in ratio of green fluorescence intensity and green fluorescence positivity of treated/infected cells to those of un-treated infected cells, respectively.

### 2.10. Plaque Reduction Assay for IC50

Monolayer of BHK-21 cells in 6-well plates was infected with JEV (100 pfu) and simultaneously treated with MJ-47 (0, 1, 10, 20, 30, 40, and 50 μM). After 60-h incubation (37 °C, 5% CO_2_), plaque was quantified after staining by naphthol blue-black dye. Inhibitory concentration showing 50% JEV plaque reduction (IC50) was calculated using plaque reduction data from three independent experiments.

### 2.11. Virucidal and Attachment Assays

In the virucidal assay, the mixture of JEV (10^4^ pfu per reaction) with indicated concentrations of MJ-47 was incubated for 1 h at 37 °C. The 100-fold dilution of the mixture was added onto a BHK-21 cell monolayer, and then followed by the protocol of plaque assay. In the virus attachment assay, the mixtures of JEV (50 pfu per well) with MJ-47 (0, 1, 10, or 50 μM) were immediately added onto a BHK-21 cell monolayer at 4 °C for 1 h. After washing with cold PBS, the cell monolayer was followed by the protocol of plaque assay. Virucidal and attachment inhibition activities were calculated as residual plaques.

### 2.12. Statistical Analysis

Each piece of data from three independent experiments was represented as mean ± standard deviation (S.D.). Each group was evaluated by a Student *t*-test. If its *p*-value was lower than 0.05, the comparison was recognized as a statistical significance.

## 3. Result

### 3.1. Functional Analysis of pBR322 Plasmid-Based JEV Replicon

A JEV-EGFP replicon consisting of five fragments self-replicated in *E. coli* and was verified using restriction endonuclease fragmentation and nucleotide sequence analyses. The sizes of resultant restriction fragments using single- and double-digestion matched the given restriction map of JEV-EGFP replicon. Moreover, nucleotide sequence analysis with sequence-specific primers (Table S2) indicated that 10 synonymous mutations and 23 nonsynonymous mutations were identified in a JEV-EGFP replicon, which was nearly identical to the JEV T1P1 parent strain by the nucleotide identity of 99.62% and amino acid identity of 99.19% (Tables S3 and S4). 

**Figure 4:**
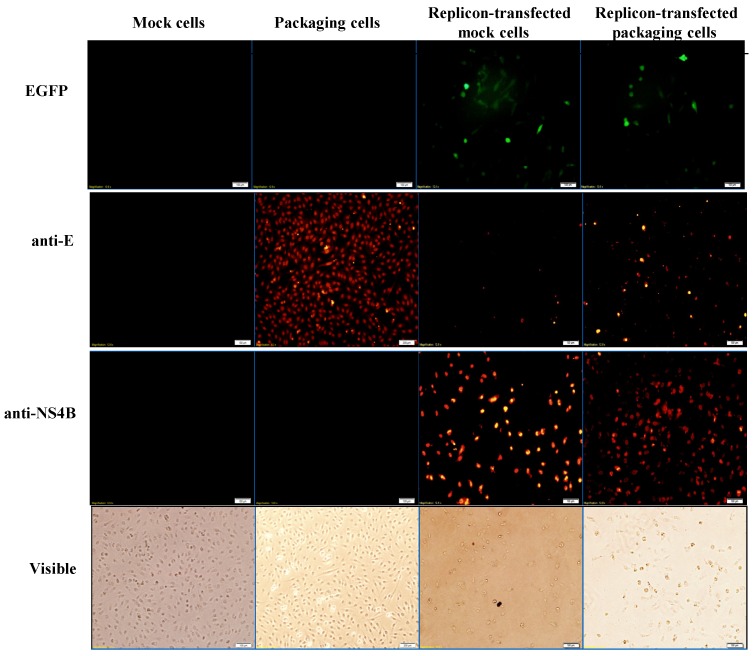
Analysis of the expression of EGFP and JEV in mock cells, packaging cells, replicon-transfected mock cells and replicon-transfected packaging cells using immunofluorescent staining. The cells were washed, fixed, and reacted with indicated primary antibodies and Alexa Fluor 546-conjugated secondary antibodies (ThermoFisher, Waltham, MA, USA). Finally, imaging of cells was taken by immunofluorescent microscopy. In addition, the cell morphology was photographed using bright-field microscopy.

To examine the functional properties of JEV-EGFP replicon in vitro, viral genome transcription and translation of the replicon in human medulloblastoma TE671 cells were further characterized (Figure 3 and Figure 4). Relative copy numbers of positive- and negative-sense JEV subgenomes were measured using real-time reverse transcription-PCR with specific capture and amplification primers, and normalized by housekeeping gene GAPDH (Figure 3 and Figure S3). The copy numbers of positive-sense JEV subgenomes were 2.3 × 10^4^ copies in transfected cells with JEV-EGFP replicon at 36 h post transfection. Meanwhile, the copy numbers of negative-sense JEV subgenomes were detectable at late stage of JEV replication in replicon-transfected cells. The ratio of positive- to negative-sense RNA synthesis was greater than 3000, implying highly efficient self-replication of viral genomes in replicon-transfected cells. In addition, fluorescent microscopy revealed the green fluorescence in the replicon-transfected cells, indicating the expression of replicon-driven EGFP. Immunofluorescent staining also demonstrated the synthesis of viral proteins like NS4B in transfected cells (Figure 4). Interestingly, the cytopathic effect (CPE) was observed in replicon-transfected mock cells and replicon-transfected packaging cells, but not in mock cells and packaging cells (Figure 4). In addition, the CPE level was higher in replicon-transfected packaging cells than replicon-transfected mock cells, which was associated with a lower amount of E and NS4B in replicon-transfected packaging cells compared to replicon-transfected mock cells. JEV non-structural proteins like NS4B were more abundant than EGFP at late stage of JEV replicon replication in mock cells. The results showed that pBR322 plasmid-based JEV replicon enabled production of the self-replicated JEV RNA genomes and viral non-structural proteins in cells. 

### 3.2. Production and Infectivity of JEV-EGFP SRIPs

To generate the packaging cell line for the production of JEV-EGFP SRIPs, a stably transfected TE671 cell line with pFlag-CMV3-CprME was selected after a long-period screening with G418. The expression of JEV structural proteins, such as E protein, in a stably transfected packaging cell line was detected using immunofluorescent staining (Figure 4). Subsequently, pBR322-based JEV-EGFP replicon was transfected into the packaging cell line. Real-time RT-PCR and immunofluorescent staining demonstrated JEV-EGFP replicon-driven viral positive- and negative-sense RNA genomes were synthesized, as well as replicon-driven EGFP and viral non-structural proteins being expressed in the packaging cell line (Figure 3 and Figure 4). The copy numbers of positive-sense JEV subgenomes were 2.9 × 10^4^ copies in packaging cells transfected with JEV replicon at 36 h post transfection. The copy numbers of negative-sense JEV subgenomes were also detectable at late stages of JEV replication in replicon-transfected packaging cells. The ratio of positive- to negative-sense RNA synthesis was greater than 6000, implying higher efficient self-replication of replicon-driven viral subgenomes in packaging cells compared to mock cells. Immunofluorescent staining indicated that the amount of viral proteins, such as E and NS4B, was lower in packaging cells transfected with JEV-EGFP replicon than un-transfected packaging cells or mock cells transfected with replicon, which was associated with the cytopathic effect in replicon-transfected packaging cells due to the generation of JEV-EGFP SRIPs. Therefore, JEV-EGFP SRIPs were harvested from the supernatant and lysate of replicon-transfected packaging cells 36 h post transfection.

**Figure 5:**
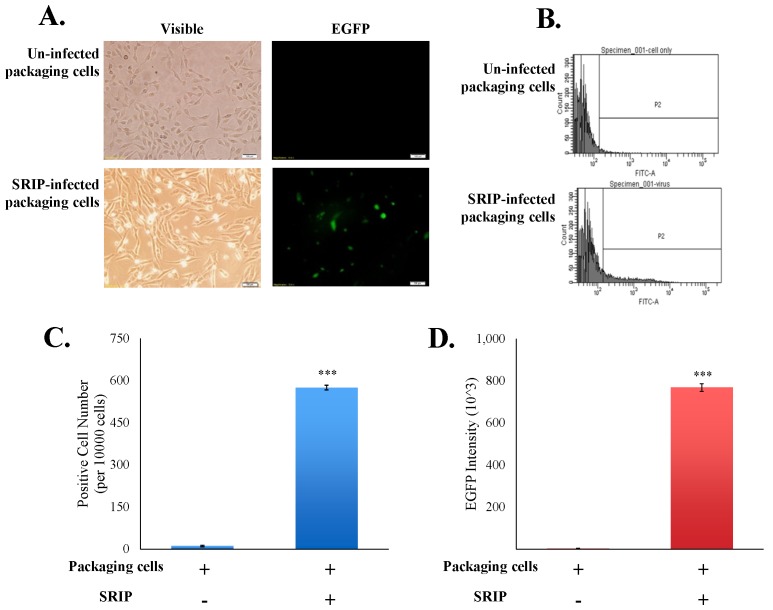
Analysis of the infectivity of JEV-EGFP SRIPs in packaging cells. The cytopathic effect and the green fluorescence of SRIP-infected cells were photographed using immunofluorescent microscopy 36 h post infection (**A**). Un-infected and infected packaging cells were harvested, washed, fixed, and then analyzed using flow cytometry with excitation at 495 nm and emission at 520 nm (**B**). EGFP-positive cells (**C**) and green fluorescent intensity (**D**) of infected cells were recorded, respectively. ***, *p*-value < 0.001 compared with un-infected packaging cells.

To examine the infectivity of JEV-EGFP SRIPs in vitro, the packaging cells were infected with JEV-EGFP SRIPs at 10 TCID_50_, and then SRIP-induced cytopathic effect and SRIP-driven EGFP in packaging cells were observed 36 h post infection (Figure 5A). The green fluorescent intensity of mock-infected and SRIP-infected cells was quantitated using flow cytometry (Figure 5B–D). SRIP-infected cells had a significant increase in the ratio of green fluorescence-positive cells and the accumulated fluorescence intensity compared to mock-infected cells (Figure 5C,D). The results demonstrated the production of infectious SRIPs in co-transfected cells and quantitative analysis of SRIP infectivity using flow cytometry. 

### 3.3. Evaluation of the Antiviral Screening Platform Using JEV-EGFP SRIPs and Flow Cytometry

**Figure 6:**
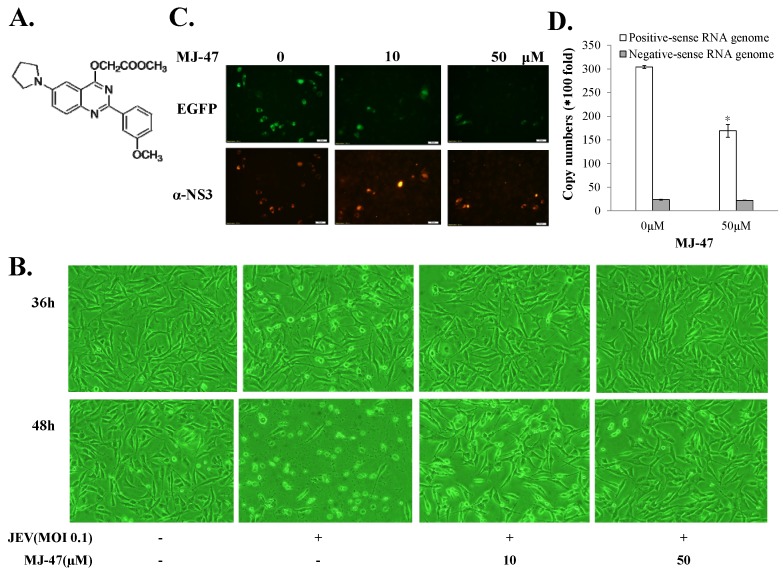
Reduction of JEV-induced cytopathic effects and viral protein expression in the presence of MJ-47. The structure of MJ-47 (methyl 2-((2-(3-methoxyphenyl)-6-(pyrrolidin-1-yl)quinazolin-4-yl)oxy)acetate) is shown in (**A**). Virus-induced cytopathic effect was photographed 36 and 48 h post treatment with MJ-47 by phase-contrast microscopy (**B**). Moreover, mock cells were infected with JEV-EGFP SRIPs at 10 TCID_50_ in the presence and absence of MJ-47. The expression of EGFP (green fluorescence) and viral proteins was examined 36 h post infection using immunofluorescent staining with primary antibodies and Alexa Fluor 546-conjugated secondary antibodies (**C**). Real-time RT-PCR analysis of positive- and negative-sense viral subgenomes in JEV SRIP-infected mock cells in the presence and absence of MJ-47 (**D**). *, *p*-value < 0.05 compared with SRIP-infected mock cells.

**Figure 7:**
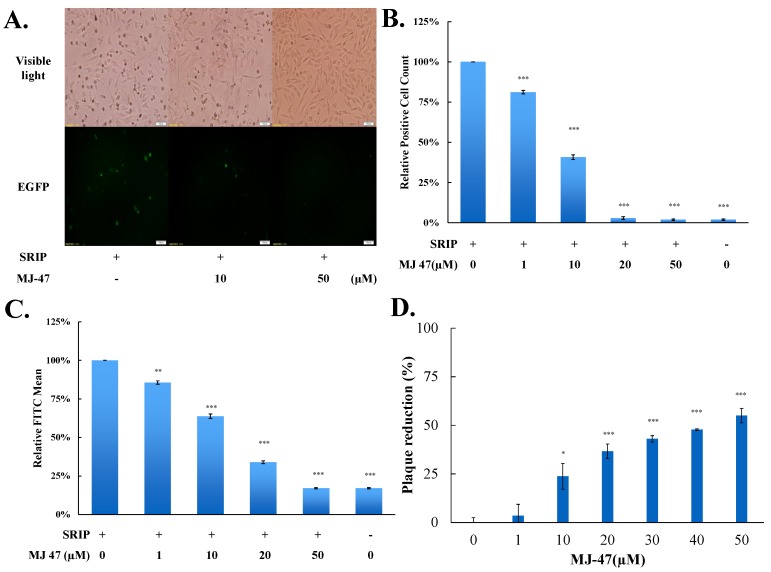
Measurement of IC50 value of MJ-47 against JEV using the system of JEVSRIP-infected packaging cells plus flow cytometry and the plaque reduction assay. Virus-induced cytopathic effect in packaging cells was photographed 36 h post SRIP infection in the presence or absence of MJ-47 by phase-contrast and fluorescent microscopies (**A**). In the system of JEV-EGFP SRIP plus flow cytometry, CprME-expressing packaging cells were infected with JEV-EGFP SRIPs at 10 TCID_50_ in presence and absence of MJ-47, harvested 36 h post infection, and analyzed using flow cytometry. The ratio of EGFP-positive cells (**B**) and relative green fluorescent intensity (**C**) in MJ-47-treated SRIP-infected cells to those in SRIP-infected cells were represented after normalizing the un-infected cells. In the plaque assay (**D**), cell monolayer was infected with JEV (100 pfu), immediately treated with MJ-47, overlaid with 2 mL of a methylcellulose medium for 3 days, and then stained with naphthol blue-black dye. Plaque reduction was calculated from ratio of experimental data to un-treated control. *, *p*-value < 0.05; **, *p*-value < 0.01; ***, *p*-value < 0.001 compared with mock control.

To access whether JEV-EGFP SRIPs could be applicable in the antiviral assays and substitute for JEV virions, a combination of JEV-EGFP SRIPs and flow cytometry was used to evaluate the IC50 values of MJ-47 compound against JEV, compared to a plaque reduction assay with wild type virions (Figure 6 and Figure 7). MJ-47 was a quinazolinone derivative with a low cytotoxicity (Figure 6A, Supplemental Figure S5). MJ-47 served as a significant inhibition on the JEV-induced cytopathic effect in TE671 cells, respectively (Figure 6B). MJ-47 had no effect on virucidal activity and virus attachment inhibition (Figure S6). Moreover, MJ-47 concentration-dependently reduced the expression of EGFP and viral proteins like NS3 in SRIP-infected mock cells. MJ-47 also suppressed the synthesis of positive-sense RNA subgenomes in SRIP-infected mock cells 36 h post treatment (Figure 6C,D), which demonstrated MJ-47 significantly decreasing the efficient self-replication of replicon-driven viral subgenomes. To examine the anti-JEV ability of MJ-47 using JEV-EGFP SRIPs, CprME-expressing packaging cells were subsequently infected with SRIPs at 10 TCID_50_ and simultaneously treated with and without MJ-47 (Figure 7). MJ-47 significantly inhibited SRIP-induced cytopathic effects and reduced the expression of green fluorescent protein in SRIP-infected packaging cells (Figure 7A). Green fluorescence in un-infected packaging cells and SRIP-infected packaging cells were analyzed using flow cytometry (Figure 7B,C). IC50 values of MJ-47 against JEV were 6.3 µM based on green fluorescence-positive cells and 16.3 µM according to the accumulated fluorescence intensity, respectively (Figure 7B,C). In comparison to JEV-EGFP SRIPs, wild type virions were used to examine the anti-JEV ability of MJ-47 in a conventional plaque assay, in which IC50 value of MJ-47 was 34.5 µM (Figure 7D). The results indicated that the antiviral assay with a combination of JEV-EGFP SRIPs and flow cytometry enabled measurement of the IC50 values of antiviral agents, and was more sensitive than the plaque reduction assay with JEV virions.

## 4. Discussion

The CMV promoter-launched replicon did not need either an in vitro transcription step required for the T7 promoter-launched replicon, or co-transfection of plasmid containing T7 RNA polymerase and T7 promoter-launched replicon [21,38]. Moreover, DNA-dependent RNA polymerase had a higher fidelity than RNA-dependent RNA polymerase. Thus, this study constructed the CMV promoter-launched JEV replicon carrying a green fluorescence reporter (EGFP) (Figure 1 and Figure 2). However, the cloning process of JEV full-length infectious clones and replicons revealed that nonsense mutations (nt C741T and nt C831T) and the deletion with nt 574–1936 appeared in the one-third (nt 1–3600) of the JEV genome at 5′-end (data not shown). Importantly, the nonsense mutations and deletion identified resulted in the production of truncated M protein and the loss of E protein in the cells. Our findings indicated that the prokaryotic promoter activity of the 5′ one-third of JEV genome directed the synthesis of the structural proteins C, prM/M and E that might be toxic genes or proteins in bacteria. The toxic sequences, especially the region encoding the prM protein, correlated with the genetic instability of JEV infectious clones and replicons in *E. coli*. Our findings were similar to previous reports that several *E. coli* promoter sequences were identified within the 5′ end of JEV genome, particularly nt 54–120 [39,40]. Since a restriction enzyme ApaI site was located with JEV genome nt 114–119, the nucleotides 1 to 119 of JEV genome encoding eight amino acids of the C protein were fused with the EGFP reporter gene in the CMV promoter-launched JEV replicon. In addition, the addition of restriction enzyme sequences NotI and ClaI before encoding partial C-terminal transmembrane (28 amino acids) of E protein allowed two unique restriction enzyme sites in the DNA-based JEV replicon that could be used as the expression vector in mammalian cells. Therefore, the CMV promoter-launched JEV-EGFP replicon did not contain the toxic sequence encoding the prM protein, allowing the stability of JEV cDNA clones in bacteria. In addition, recombinant plasmid with the nucleotide sequence 96–2477 of JEV genome encoding structural proteins C, prM/M and E was stable in bacteria due to lacking of the prokaryotic promoter at nt 54–120. The pFlag-CMV-CprME exhibited the plasmid stability in *E. coli*.

The pBR322 plasmid, a low copy number plasmid, was chosen as the JEV replicon vector. The plasmid pBR322-based JEV-EGFP replicon was genetically stable in *E. coli*, exhibiting high nucleotide (99.62%) and amino acid (99.19%) identities (Tables S3 and S4). The pBR322-based replicon also had a higher yield in *E.coli* than cosmid vectors and bacterial artificial chromosomes [21], offering an effective and efficient approach for manipulating and cloning the viral genes. Among 23 non-silent mutations in the replicon compared to the original genome sequence of JEV T1P1 strain, eight mutated residues appeared within NS5 and three mutations occurred within NS3 (Table S4). These eight mutations in the replicon did not relate with active sites and conserved motifs of NS3 protease and helicase as well as NS5 methyltransferase and RNA-dependent RNA polymerase based on the structure and functionality analysis of flaviviral non-structural proteins [41,42]. In addition, the other 12 amino acid substitutions in the replicon sporadically existed within NS1, NS2A, NS2B, NS4A and NS4B did not match with the reported mutation sites that altered JEV replication [43,44]. The finding suggested that 23 mutations in the replicon might have no significant effect on virus replication. Functional characterization of JEV-EGFP replicon indicated a significant increase of green fluorescence intensity in replicon-transfected cells (Figure 4). Moreover, the percentage and yellow fluorescent intensity of NS4B-positive cells were is much higher than the percentage and green fluorescent intensity of EGFP-expressing cells post transfection with JEV-EGFP replicon. The results might be associated with the loss or quenching effect of EGFP fluorescence by the fixation process of stained cells with paraformaldehyde, ethanol or methanol [45]. Moreover, the synthesis of positive- and negative-sense RNA subgenomes as well as the expression of JEV proteins were detected in replicon-transfected cells (Figure 4 and Figure 5). JEV-EGFP replicon contained CMV immediate-early promoter at the 5′-end, as well as HDVr and SV40 termination and polyadenylation sequences at the 3′-end, which allowed the production of functional JEV subgenomes (Figure 2, Figure 3 and Figure 4). The pFlag-CMV-CprME was utilized to establish the packaging cell line that continuously expressed C, prM/M, and E proteins to allow the production of JEV SRIPs 36 h post a transient transfection with JEV-EGFP replicon (Figure 3, Figure 4 and Figure 5). Therefore, producing JEV SRIPs was an alternative approach to overcoming the obstacles in the genetic instability of JEV infectious clones using the full-length cDNA sequence. Although there is still a question about whether these mutations affect the genome replication efficiency, JEV-EGFP replicon and SRIP generated in this study could be used to screen antiviral agents. Then, the antiviral activity of identified agents might be further confirmed using wild type JEV virions.

EGFP reporter was the real-time functional marker as the genome replication and viral protein expression of JEV-EGFP replicon in mock cells and SRIP in packaging cells, detected using fluorescent microscope (Figure 3, Figure 4 and Figure 5). The fluorescent intensity and positivity in SRIP-infected cells were further quantitated using flow cytometry (Figure 5). The reasons for low EGFP-positive percentage and fluorescent intensity in un-treated SRIP-infected cells could be (1) a low infectious dose with 10 TCID_50_ SRIPs; (2) the loss of EGFP fluorescence with the fixation with ethanol, and (3) a highly stringent threshold for the positivity of green fluorescence in the infected cells. For increasing the EGFP-positive cells without treatment, the elevation of MOI was used in the assay and the fluorescent intensity in the infected cells were directly measured using a fluorescence microplate reader under the condition without the fixation agents. In the screening assay to identify antiviral agents, Figure 7C based on the total fluorescent intensity showed the near line with a negative slope in antiviral activity of MJ-47 against JEV, but not Figure 7B based on the green fluorescence positivity. Since plaque reduction assay was labor-intensive, time-consuming and safe keeping, the combination of JEV-EGFP SRIP and flow cytometry might be easy, rapid, and safer approaches to determining the IC50 values of antiviral agents. Comparison of IC50 values of MJ-47 against JEV using plaque reduction and JEV-EGFP SRIP plus flow cytometry indicated the IC50 value (6.28~16.05 µM) of MJ-47 by JEV-EGFP SRIP plus flow cytometry was lower than plaque reduction assay (34.5 µM) (Figure 7). The result demonstrated that the assay of JEV-EGFP SRIP plus flow cytometry was more sensitive than plaque reduction assay, showing the potential application in antiviral drug screening and neutralizing assays. In addition, the system of JEV-EGFP SRIP in packaging cells plus flow cytometry was easily observed using a fluorescent microscope and quantitated by flow cytometry, suggested as a better tool compared to the luciferase reporter system [38,40]. The approach for the design of JEV-EGFP SRIP plus flow cytometry might be applicable to other flaviviruses, such as dengue and Zika viruses. Moreover, the alternative approaches to quantitate replicon-driven reporter activity might be applicable for high-throughput screening antiviral agents, such as the combination of JEV-EGFP SRIP and fluorescence microplate reader. 

MJ-47 (methyl 2-((2-(3-methoxyphenyl)-6-(pyrrolidin-1-yl)quinazolin-4-yl)oxy)acetate), a quinazolinone derivative, processed the potently anti-JEV activity, in which an IC50 value of 6.28 µM was determined by the assay of JEV-EGFP SRIP in packaging cells plus flow cytometry (Figure 7). Moreover, MJ-47 reduced the synthesis of viral genomes and the expression of viral proteins (Figure 6C,D), implying that MJ-47 significantly decreased the efficient self-replication of replicon-driven viral subgenomes. However, MJ-47 affected neither irreversible loss of JEV infectivity (virucidal activity), nor virus attachment to host cells (Supplemental Figure S6). Therefore, we suggested that anti-JEV mechanism(s) of MJ-47 could be related with the influence on late stages of JEV replication cycle, e.g., viral transcription and translation. Interestingly, several quinazolinone derivatives had identified their antiviral activity against a broad range of DNA and RNA viruses, such varicella-zoster virus, cytomegalovirus, influenza A virus, and hepatitis C virus [46,47,48]. A 2-pyridinyl-4(3H)-quinazolinone derivative potently inhibited the replication of influenza A virus in vitro via suppressing virus neuraminidase and cellular NF-κB signal [47]. Quinazolinone-containing macrocycles were considered as next-generation HCV NS3/4a protease inhibitors [48]. Therefore, MJ-47’s antiviral ability might be further examined against other flaviviruses. 

## 5. Conclusions

In conclusion, the JEV-EGFP replicon, avoiding genetic instability of the 5′-end one-third of the full-length JEV genome cDNA, was constructed into the low-copy plasmid pBR322, regulated under the control of CMV promoter, and transfected into a packaging cell line expressing viral structural proteins for the generation of JEV-EGFP SRIPs. The reporter gene EGFP, correlating with the infectivity of JEV-EGFP SRIPs in packaging cells, were easily and rapidly utilized to screen antiviral agents using fluorescent microscopes. The combination of JEV-EGFP SRIP plus flow cytometry was a reliable, effective, and efficient assay for measuring the IC50 values of antiviral agents like MJ-47 based on the fluorescent intensity and positivity of SRIP-infected packaging cells post treatment.

## Supplementary Materials

The following are available online at www.mdpi.com/1999-4915/9/4/76/s1, Figure S1: Modification of multiple cloning sites by the insertion of the linker KpnI-NotI-XhoI into the pBR322 with EcoRI and BamHI double digestion, Figure S2: Preparation of chimeric sequences of CMV promoter and JEV 5’-seq (1-200 nt) using jumping PCR, Figure S3: Primer design for quantitative analysis of positive- and negative-sense RNA genome using SYBR-Green RT-PCR, Figure S4: General experimental procedures for MJ-47 synthesis. NaH, sodium hydride; DMF, N,N-dimethylformamide, Figure S5: Survival rate of treated TE671 cells with MJ-47. TE671 cells cultured on 96-well plates were treated with MJ-47, incubated for 48 h, and then followed by MTT assay, Figure S6: Virucidal and attachment inhibitory activities of MJ-47 against JEV, Table S1: Primers for constructing pBR322-based JEV-EGFP replicon and pFlag-CMV-CprME plasmid, Table S2: Primers for sequencing pBR322-based JEV-EGFP replicon and pFlag-CMV-CprME plasmid, Table S3: List of synonymous substitutions in the JEV-EGFP replicon, Table S4: List of nonsynonymous substitutions in the JEV-EGFP replicon.
