# Supplementary material for: Single-Round Infectious Particle Antiviral Screening Assays for the Japanese Encephalitis Virus
Source: Viruses. 2017 Apr 10;9(4):76. doi: 10.3390/v9040076 (PMC5408682; doi:10.3390/v9040076)
Supplement: Supplementary file 1 [file viruses-09-00076-s001.pdf]

Supplementary Materials: Single-Round Infectious Particle Antiviral Screening Assays for the Japanese Encephalitis Virus

Chien-Yi Lu, Mann-Jen Hour, Ching-Ying Wang, Su-Hua Huang, Wen-Xiang Mu, Yu-Chun Chang and Cheng-Wen Lin

Table S1. Primers for constructing pBR322-based JEV-EGFP replicon and pFlag-CMV-CprME plasmid.

Fragments	Primer Sequences	Primer Location	Restriction Sites/Extra Sequence	Templates
1				
Forward<br>Reverse	5′-GCGC**GGTACC**TAGTAATCAATTACGGGG-3′<br>5′-CCAAGAAGTTCACACAGATAAACTTCTACGGTT CACTAAACGAGCTCTGCTTATATAGACCTC-3′	nt 1-18 *<br>nt 538-600 *	KpnI	pcDNA3.1/ HisC
2				
Forward<br>Reverse	5′-AGAAGTTTATCTGTGTGAACTT-3′<br>5′-TCATTACTACCCTCTTCACTC**GCGGCCGC**TC-3′	nt 574-595 *<br>nt 673-700 *	NotI	JEV genomic cDNA
3				
Forward<br>Reverse	5′-TAAT**GGGCCC**ATGGTGAGCAAGGGCGAGGAG-3′<br>5′-CT**GCGGCCGC**TGGCCCAGGGTTGGACTCAACG TCTCCTGCCAACTTGAGAAGGTCAAAATTCTTGTAC AGCTCGTCCATGCC-3′	nt 687-713 *<br>nt 1398-1468 *	ApaI<br>NotI/FMDV 2A	pEGFP-N1
4				
Forward<br>Reverse	5′-GGGC**CTCGAG**CGAAATGACCGACCAAGCGA-3′<br>5′-GGCC**GGATCC**TGCAGTGAAAAAAATGCTTT-3′	nt 10150-10175 *<br>nt 10378-10403 *	XhoI<br>BamHI	pcDNA3.1/ HisC
5				
Forward	5′-CA**GCGGCCGC**AGGAGACTACAAAGACGATGA CGACAAGGGCATCGATGTCAACGCACGAGACCGA TCAATTGCCAGCGGCCGCAGGAGACTACAAAGAC GATGACGACAAGGGC**ATCGAT**GTCAACGCACGAG ACCGATCAATTGC-3′	nt 1459-1602 *	NotI/Flag tag + ClaI	JEV genomic cDNA
Reverse	5′-CGCG**CTCGAG**CTTCTCCCTTAGCCTACCGAAGT AGCCCAGGTCGGACCGCGAGGAGGTGGAGATGCC ATGCCGACCCGTGTTCTTCCTCACCACCAGCTACA-3′	nt 10058-10155 *	XhoI/HDVr	
CprME				
Forward<br>Reverse	5′-GC**GAATTC**AATGACTAAAAAACCAG-3′<br>5′-CC**TCTAGA**TTCAAGCATGCACATTGGTC-3′	nt 94-110 **<br>nt 2458-2477 **	EcoRI<br>XbaI	JEV genomic cDNA

* Nucleotide number based on JEV-EGFP replicon; ** Nucleotide number based on JEV-T1P1 strain cDNA.

Table S2. Primers for sequencing pBR322-based JEV-EGFP replicon and pFlag-CMV-CprME plasmid.

Primers	Primer Sequences	Primer Location *
**JE1F**	5′-AGAAGTTTATCTGTGTGAACTT-3′	nt 1-22
**JE123R**	5′-TACCGGGCCCTCCTGGTTTT-3′	nt 123-104
**JE200R**	5′-TCATTACTACCCTCTTCACTC-3′	nt 201-181
**JE(795-821)F**	5′-AAAAAAGAGGCTTGGCTGGATTCCACG-3′	nt 795-821
**JE(821-795)R**	5′-CGTGGAATCCAGCCAAGCCTCTTTTTT-3′	nt 821-795
**JF2975R**	5′-CACGGGTTGATGTGATGCCAAA-3′	nt 2076-2055
**JE2016F**	5′-GACATGACCCCCGTTGGGC-3′	nt 2016-2034
**JE2063R**	5′-CGCGACGAAGGGGTTCAC-3′	nt 2063-2046
**JE2412F**	5′-TCAATTGCTTTGGCCTTCTTA-3′	nt 2412-2432
**JE2584R**	5′-GGCAAATATTTATACCTATC-3′	nt 2584-2565
**JE2948F**	5′-TTCGGCTTTGGCATCACATCA-3′	nt 2498-2518
**JE4464R**	5′-CTCCGTCATCATCCAGTTTCAC-3′	nt 4464-4455
**JE4307F**	5′-ACCCTTCATGCTGGCAGGTC-3′	nt 4307-4326
**JE5000F**	5′-GCGAGGAACATCCGGCTCACC-3′	nt 5000-5020
**JE5200F**	5′-CAGGGAAAACCAGGAAAATT-3′	nt 5200-5219
**JE5776R**	5′-GGACTTGCGGTTGAGTTGGATG-3′	nt 5776-5755
**JE5685F**	5′-TGTGGTTTGTGGCGAGCGTAAA-3′	nt 5685-5706
**JE6400F**	5′-ATGCAAGAGTTTATGCAGATC-3′	nt 6400-6420
**JE7220R**	5′-CCAACAGCCAAGGAAGACGAG-3′	nt 7220-7200
**JE7200F**	5′-CTCGTCTTCCTTGGCTGTTGG-3′	nt 7200-7220
**JE7800F**	5′-GTGGACCGCACTGAAGCACGCA-3′	nt 7800-7821
**JE8306F**	5′-CTGTCCCGAAACTCCAATCACG-3′	nt 8306-8327
**JE8800F**	5′-TTGTGCACCAAGGAAGAATTCA-3′	nt 8800-8821
**JE8909R**	5′-CTGTTCAGCGAACACTGCTCCA-3′	nt 8909-8888
**JE9431F**	5′-TGCAGCAGAAGGAAAGACCGTGAT-3′	nt 9431-9454
**JE10100F**	5′-GATGACCACAGAAGACATGC-3′	nt 10100-10119
**JE10000R**	5′-CTACGATGGAAGTATAGGAG-3′	nt 10000-9981
**JE10600F**	5′-CGGAAGCAGGTCCCTGCTCACT-3′	nt 10600-10621
**JE10975R**	5′-AGATCCTGTGTTCTTCCTCAC-3′	nt 10975-10955

* Nucleotide number based on JEV-T1P1 strain cDNA.

Table S3. List of synonymous substitutions in the JEV-EGFP replicon.

No.	Site(nt)	Protein Site	Sequence	Amino Acid
1	JEV 4482-4484	NS2B	GAC→GAT	90D
2	JEV 4689-4691	NS3	AGG→AGA	28R
3	JEV 4980-4982	NS3	GCT→GCC	125A
4	JEV 5370-5372	NS3	AAT→AAC	255N
5	JEV 6015-6017	NS4A	GGA→GGG	6G
6	JEV 7299-7301	NS4B	CAA→CAG	132Q
7	JEV 7680-7682	NS5	AGA→AGG	9R
8	JEV 8562-8564	NS5	AAA→AAG	303K
9	JEV 9327-9329	NS5	AAT→AAC	558N
10	JEV 9930-9932	NS5	AAT→AAC	759N

Table S4. List of nonsynonymous substitutions in the JEV-EGFP replicon.

No.	Site(nt)	Protein Site	Sequence	Amino Acid
1	JEV 3183-3185	NS1	GTT→GCT	V236A
2	JEV 3345-3347	NS1	GAT→GGT	D290G
3	JEV 3882-3884	NS2A	GCC→ACC	A113T
4	JEV 4188-4190	NS2A	ATG→ACG	M215T
5	JEV 4365-4367	NS2B	GAT→GAG	D51E
6	JEV 4389-4391	NS2B	GAC→GGC	D59G
7	JEV 4971-4973	NS3	AAG→GAG	K122E
8	JEV 5244-5246	NS3	ATC→ACC	I213T
9	JEV 5820-5822	NS3	TTT→TCT	F405S
10	JEV 6057-6059	NS4A	AGT→GGT	S20G
11	JEV 6198-6200	NS4A	AAG→AGG	K67R
12	JEV 6255-6257	NS4A	AAG→ATG	K86M
13	JEV 6594-6596	NS4A	CTC→TTC	L199F
14	JEV 7080-7082	NS4B	AAG→GAG	K59E
15	JEV 7341-7343	NS4B	GGA→AGA	G146R
16	JEV 7920-7922	NS5	TGT→CGT	C89R
17	JEV 8022-8024	NS5	ATG→GTG	M123V
18	JEV 8907-8909	NS5	CTT→CCT	L418P
19	JEV 8964-8966	NS5	GAC→GGC	D437G
20	JEV 9927-9929	NS5	TGT→TGG	C758W
21	JEV 10113-10115	NS5	AAC→GAC	N820D
22	JEV 10134-10136	NS5	AAA→AGG	K827R
23	JEV 10257-10259	NS5	AAA→AGA	K868R

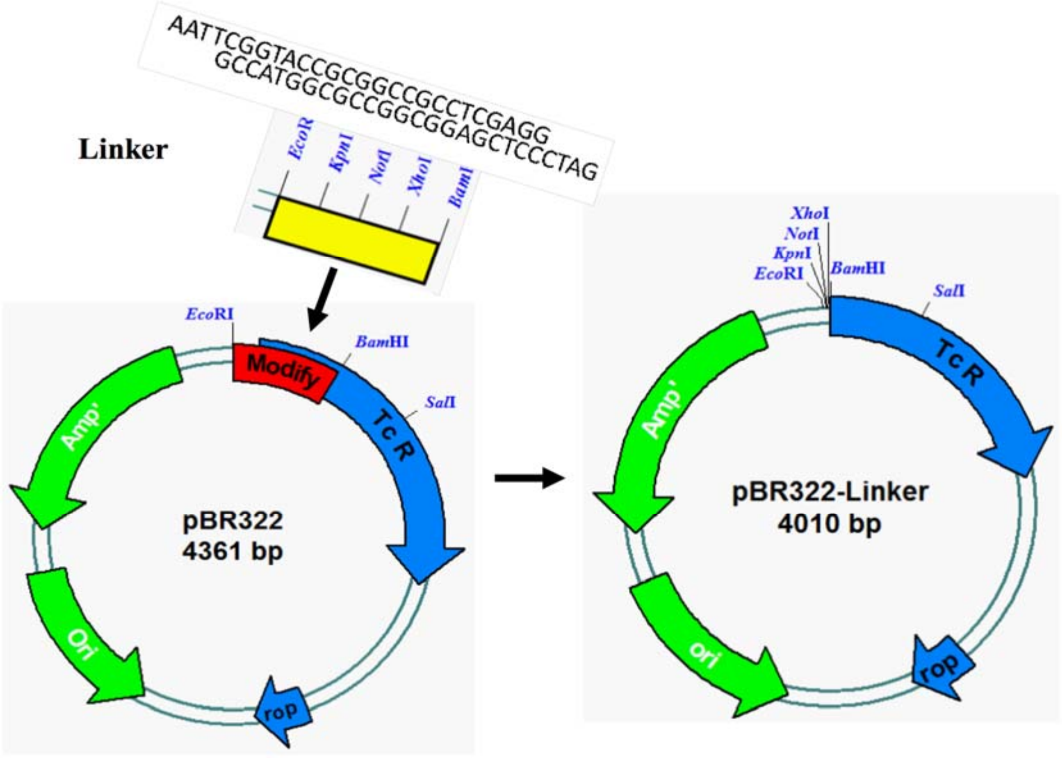

Figure S1. Modification of multiple cloning sites by the insertion of the linker KpnI-NotI-XhoI into the pBR322 with EcoRI and BamHI double digestion.

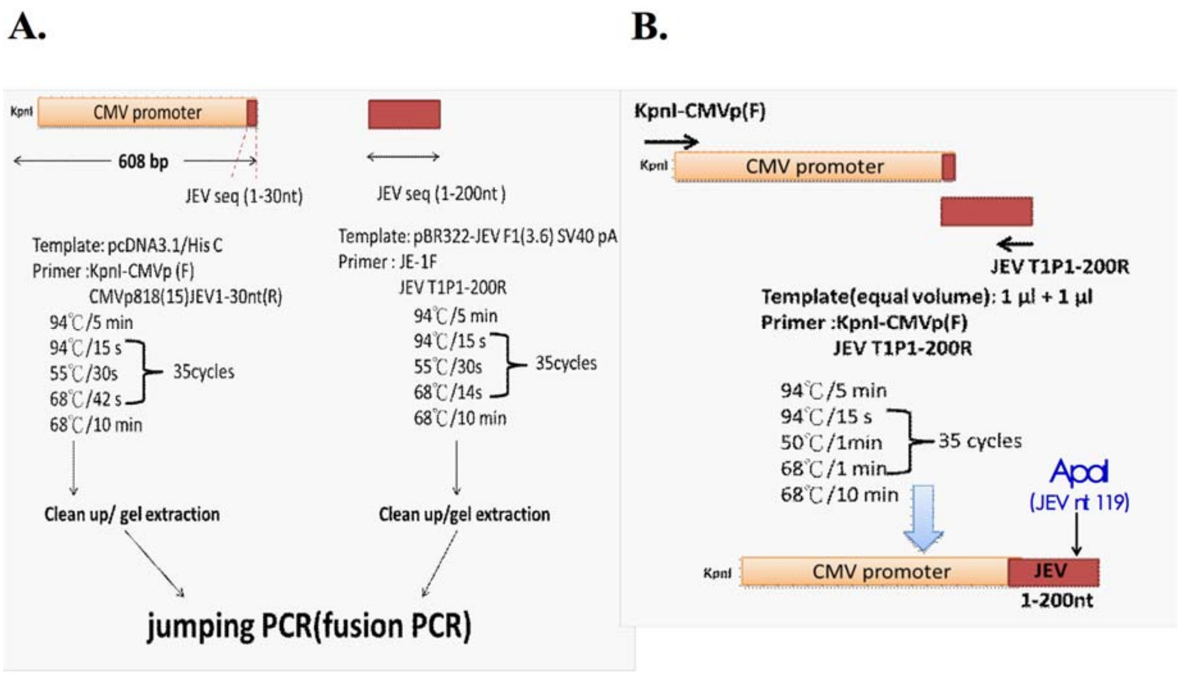

Figure S2. Preparation of chimeric sequences of CMV promoter and JEV 5′-seq (1-200 nt) using jumping PCR. Fragments 1 and 2 were amplified using PCR with the templates pcDNA3.1-HisC and JEV genomic cDNA, respectively (A). The in-frame fusion of Fragments 1 and 2 were performed using jumping PCR (B).

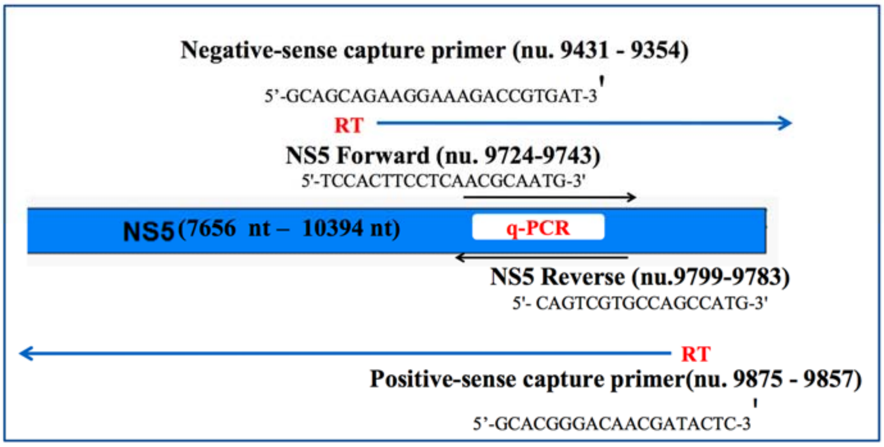

Figure S3. Primer design for quantitative analysis of positive- and negative-sense RNA genome using SYBR-Green RT-PCR.

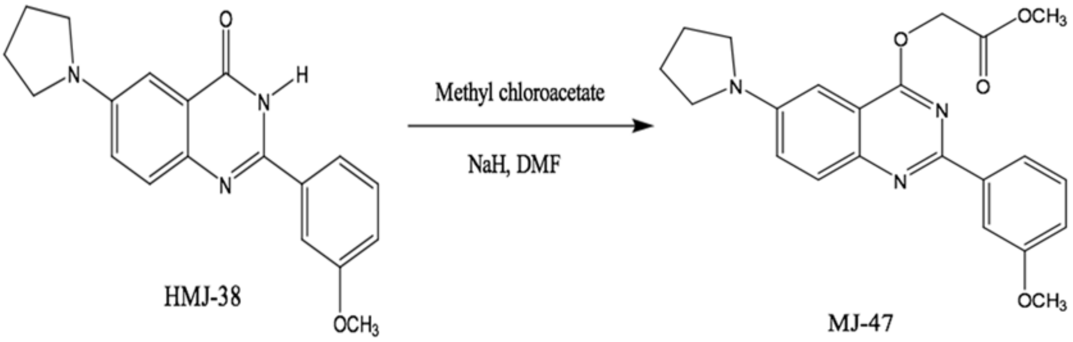

Figure S4. General experimental procedures for MJ-47 synthesis. NaH, sodium hydride; DMF, N,N-dimethylformamide**.**

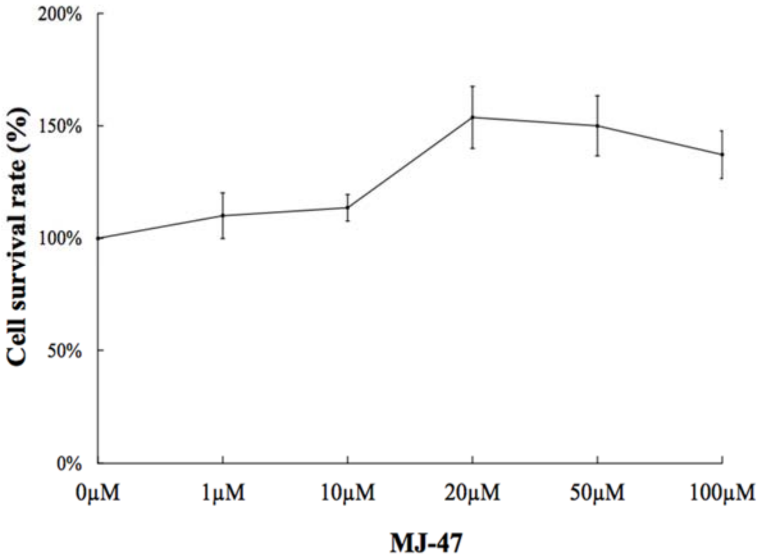

Figure S5. Survival rate of treated TE671 cells with MJ-47. TE671 cells cultured on 96-well plates were treated with MJ-47, incubated for 48 h, and then followed by MTT assay. Survival rates of cells were calculated as the ratio of OD570–630 nm of treated cells to OD570–630 nm of untreated cells.

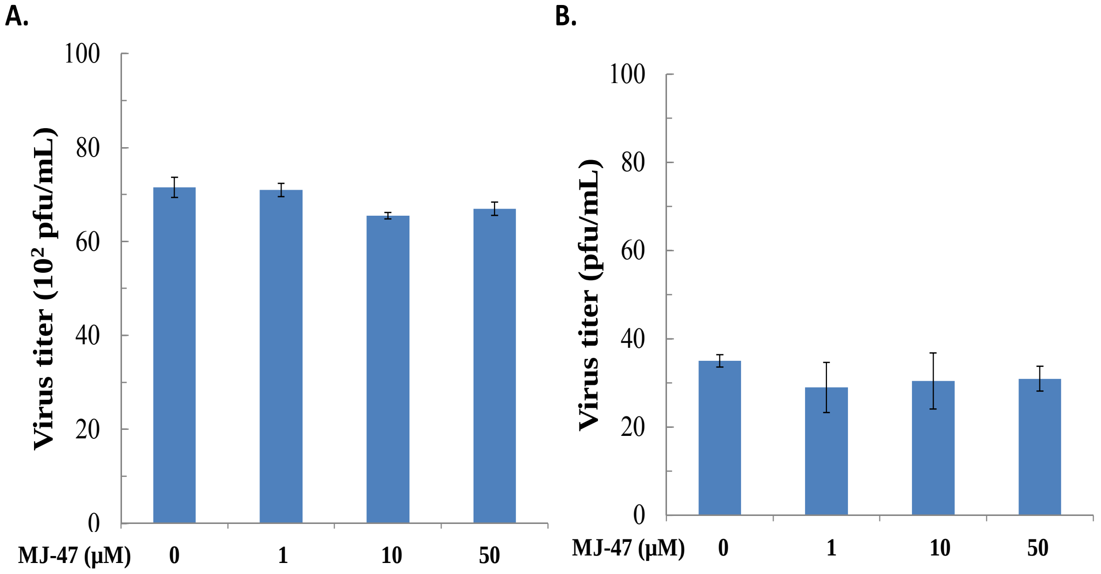

Figure S6. Virucidal and attachment inhibitory activities of MJ-47 against JEV. In a virucidal assay (A), JEV (104 pfu) was mixed with MJ-47, then incubated at 37 °C for 1 h; 100-fold dilution of the mixture was used to determine the residual infectivity using plaque assay. In the attachment assay (B), JEV (50 pfu) was mixed with MJ-47, and then immediately added onto the BHK-21 cell monolayer. After 1-h incubation, cell monolayer was washed twice with PBS, and then overlaid with 2 mL of a methylcellulose medium for three days at 37 °C in a CO2 incubator. Attachment inhibition was calculated as residual plaques.

© 2017 by the authors. Licensee MDPI, Basel, Switzerland. This article is an open access article distributed under the terms and conditions of the Creative Commons by Attribution (CC-BY) license (http://creativecommons.org/licenses/by/4.0/).
